# An epigenome-wide analysis of socioeconomic position and tumor DNA methylation in breast cancer patients

**DOI:** 10.1186/s13148-023-01470-4

**Published:** 2023-04-26

**Authors:** Jianhong Chen, Mark D. Long, Sirinapa Sribenja, Sung Jun Ma, Li Yan, Qiang Hu, Song Liu, Thaer Khoury, Chi-Chen Hong, Elisa Bandera, Anurag K. Singh, Elizabeth A. Repasky, Elizabeth G. Bouchard, Michael Higgins, Christine B. Ambrosone, Song Yao

**Affiliations:** 1 Department of Cancer Prevention and Control, Roswell Park Comprehensive Cancer Center, Buffalo, NY 14263 USA; 2 Department of Biostatistics and Bioinformatics, Roswell Park Comprehensive Cancer Center, Buffalo, NY USA; 3 Department of Molecular and Cellular Biology, Roswell Park Comprehensive Cancer Center, Buffalo, NY USA; 4 Department of Radiation Oncology, Roswell Park Comprehensive Cancer Center, Buffalo, NY USA; 5 Department of Pathology and Laboratory Medicine, Roswell Park Comprehensive Cancer Center, Buffalo, NY USA; 6 Cancer Prevention and Control Program, Rutgers Cancer Institute of New Jersey, The State University of New Jersey, New Brunswick, NJ USA; 7 Department of Immunology, Roswell Park Comprehensive Cancer Center, Buffalo, NY USA

**Keywords:** DNA methylation, Breast cancer, Socioeconomic position, Household income, *NNT*, *GPR37*

## Abstract

**Background:**

Disadvantaged socioeconomic position (SEP), including lower educational attainment and household income, may influence cancer risk and outcomes. We hypothesized that DNA methylation could function as an intermediary epigenetic mechanism that internalizes and reflects the biological impact of SEP.

**Methods:**

Based on tumor DNA methylation data from the Illumina 450 K array from 694 breast cancer patients in the Women’s Circle of Health Study, we conducted an epigenome-wide analysis in relation to educational attainment and household income. Functional impact of the identified CpG sites was explored in silico using data from publicly available databases.

**Results:**

We identified 25 CpG sites associated with household income at an array-wide significance level, but none with educational attainment. Two of the top CpG sites, cg00452016 and cg01667837, were in promoter regions of *NNT* and *GPR37,* respectively, with multiple epigenetic regulatory features identified in each region. *NNT* is involved in β-adrenergic stress signaling and inflammatory responses, whereas *GPR37* is involved in neurological and immune responses. For both loci, gene expression was inversely correlated to the levels of DNA methylation. The associations were consistent between Black and White women and did not differ by tumor estrogen receptor (ER) status.

**Conclusions:**

In a large breast cancer patient population, we discovered evidence of the significant biological impact of household income on the tumor DNA methylome, including genes in the β-adrenergic stress and immune response pathways. Our findings support biological effects of socioeconomic status on tumor tissues, which might be relevant to cancer development and progression.

**Supplementary Information:**

The online version contains supplementary material available at 10.1186/s13148-023-01470-4.

## Introduction

There has been growing public and research interest in the deleterious effects of psychosocial stress on human health, including associations with cancer incidence and mortality [1]. As lower socioeconomic position (SEP) can bring about challenges such as financial hardship, individuals from the lower end of the socioeconomic spectrum might more likely experience chronic psychosocial stress, which can lead to physiological and biological changes [2, 3].

One biological process that may be responsive to psychosocial stressors is DNA methylation. As one of the most well-studied epigenetic mechanisms, DNA methylation modulates regional gene expression and activity by adding or removing the methyl group at the CpG dinucleotides [4, 5]. Prior studies reported that DNA methylation was modifiable by various external exposures, such as cigarette smoking [6], body mass index and waist circumference [7], and nutrients of maternal diet [8]. Although the magnitude of DNA methylation changes associated with these factors is generally smaller than the differences observed between breast tumor vs. normal tissues or estrogen receptor (ER) positive vs. negative breast tumors, such epigenetic changes may still confer biologically and clinically significant effects. Moreover, the plasticity and dynamic changes of DNA methylation in response to various exposures make it a relevant intermediary mechanism linking exposures to internal biological effects. In recent years, there has been growing research interest in this burgeoning field of socioepigenomics that focuses on how psychosocial stressors get “under the skin” through epigenetic mechanisms. In an earlier study, it was found that the most socioeconomically disadvantaged group had 17% lower global DNA methylation content in blood than the least disadvantaged group [9]. Occupation has also been associated with DNA methylation, and one study found that workers doing manual labor had 24% lower levels of DNA methylation in blood samples than workers not doing manual labor [9]. Recently, several studies examined life-course SEP with DNA methylation of genes involved in inflammation and stress reactivity and found some genes were hypermethylated in individuals of low SEP [10–18].

Notably, most published socioepigenomics studies used DNA derived from peripheral blood [9–17]. Interpreting these results for relevance to solid tumors is challenging because of tissue specificity of DNA methylation and uncertain correlations between systemic and local effects of psychosocial stressors. Here, we conducted an epigenome-wide analysis of two of the commonly assessed SEP factors, total household income and educational attainment, using tumor DNA methylation data from a large population of breast cancer patients.

## Methods and materials

### Patient population

Data and breast tumor tissue samples were from participants in the Women’s Circle of Health Study (WCHS), a case–control study designed to investigate risk factors for aggressive breast cancer in Black and White women. Details on study design and participant recruitment have been described previously [19, 20]. In brief, Black and White women with primary, histologically confirmed invasive breast cancer or ductal carcinoma in situ, ages 20–75 years, were recruited and interviewed from two sites, one in NYC based at Mount Sinai School of Medicine (MSSM), and one in NJ based at The Cancer Institute of New Jersey (CINJ). Data and biospecimens were sent to Roswell Park Comprehensive Cancer Center (RPCCC) in Buffalo, NY, for processing and storage. Controls were frequency matched to cases on age and race and enrolled initially by random digital dialing and later through community health events. In-home interviews were conducted to obtain data on known and suspected risk factors for breast cancer, including educational attainment and total household income. As part of the informed consent, > 95% participants signed a release for their pathology reports and archived specimens in form of formalin-fixed, paraffin-embedded (FFPE) tumor blocks, which were obtained from the pathology departments of the treating hospitals. Data on tumor pathological features, including estrogen receptor (ER) status, were extracted from the pathology reports. This study protocol was approved by Institutional Review Boards (IRBs) at all participating institutes.

### DNA methylation assay and bioinformatic processing

DNA was extracted from FFPE breast tumor tissues as previously described [21]. Fragmented DNA was repaired using the Infinium HD DNA Restoration kit before bisulfite conversion. Real-time PCR assays using Illumina Infinium HD FFPE QC Kit (Infinium HD FFPE QC Assay Protocol, Illumina) were used for quality control (QC) assay of FFPE samples. The quality cycle threshold (QCT) value was calculated by subtracting the average Cq of Illumina QC standard from the average Cq value determined for each FFPE sample. Illumina recommends that a QCT value ≤ 5 be utilized for optimal assay performance.

Genome-wide DNA methylation analysis was performed by Roswell Park Genomics Shared Resource (GSR) using the Illumina Infinium humanMethylation450 BeadChip platform according to the manufacturer’s protocol. To minimize batch effects, DNA samples were randomized across plates according to age, ancestry, ER status, and FFPE sample type (slide, punch, or curl) using the OSAT program. The methylation level of each CpG site, expressed as β value, ranged from 0 (unmethylated) to 1 (methylated). The 450 K array data were subjected to rigorous sample and locus-specific quality control criteria, SWAN normalization, and correction for batch effects using the ComBat algorithm [22]. Low-quality probes (probes with detection *p* value > 0.05 in more than half of samples) and samples with poor detection *p* values (samples with detection *p* values < 1 × 10–5 at more than 75% of CpG loci) were removed using the IMA package [23]. We used Bowtie 2 for sequence alignment [24]. Probes known to map ambiguously and that contain single nucleotide polymorphisms were also removed, leaving the final dataset containing 276,108 CpG loci in 694 tumor samples for final analyses [25, 26].

### Annotation to regions around CpG sites and analysis of publicly available breast cancer cohort data (TCGA-BRCA)

Genomic and epigenomic context of each CpG site was visualized using the UCSC genome browser. The vertebrate conservation tracks and the transcription factor binding site (TFBS) tracks were surveyed using the UCSC phastCons base-wise conservation database and the UCSC HMR Conserved Transcription Factor Binding Site database, respectively. DNaseI hypersensitive sites indicative of open chromatin structure and transcriptional activity were extracted from ENCODE reference epigenomes. Chromatin-based features were similarly extracted from the Roadmap and ENCODE Project, and inferred chromatin states (ChromHMM) were derived from histone modification profiles. To examine the associations of DNA methylation and gene expression, publicly available RNA-seq and HM450 DNA methylation data from 780 breast cancer patients in The Cancer Genome Atlas (TCGA) were analyzed.

### Statistical analysis

Educational attainment and total household income, which are commonly collected in epidemiological studies, were used to capture SEP in the analysis. Log-transformed beta-value of each CpG probe passing the QC filters was analyzed with education and income in linear model using *limma* R package. The following factors were included in multivariable regression model: age at diagnosis, self-reported race, and tumor ER status. We also added body mass index (BMI), smoking, breastfeeding, menopausal status, and first-degree family history of breast cancer into the model, which did not substantially change the results. Thus, the parsimonious models were used. For sensitivity analysis, we also included recruiting site in the regression model.

Total household income and educational attainment levels were first modeled dichotomously (income below vs. above $50,000; education below vs. above college). For CpG probes that were significant at an array-wide level (*p* value < 1.8e−7 after Bonferroni correction for testing 276,108 CpG loci), the two SEP factors were further categorized into a 5-level variable (income: 1, $20,000 and below, 2, between $20,000 and $34,999, 3, between $35,000 and $49,999, 4, between $50,000 and $89,999, 5, $90,000 and above; education: 1, below high school, 2, high school or equal, 3, college, 4, college graduate, 5, postgraduate and above) and re-analyzed for dose–response relationships. Subgroup analyses were performed after stratification by race and tumor ER status. Aside from the epigenome-wide analysis, a *p* value threshold of 0.05 was used for statistical significance. All analyses were performed using R v4.1.1.

## Results

### Patient population

Table 1 summarizes the demographic and socioeconomic characteristics of the study population with tumor DNA methylation data stratified by family income levels, including 245 cases in low family income group (≤ $50,000) and 314 cases in relative higher family income group (> $50,000). While no differences in first-degree family history of breast cancer (with first-degree family history 16.3% vs 19.4%, *p* value = 0.34) or ER status distribution (ER-negative status 25.3% vs. 21.3%, *p* value = 0.27) were found between the two income groups, low family income group had higher BMI (29.6 vs. 27.5 kg/m^2^, *p* value = 0.001), higher prevalence of obesity (46.6% vs. 31.6%, *p* value < 0.001), slightly older age at diagnosis (52.8 vs. 50.5 years, *p* value = 0.01), less cases in menopausal status (44.1% vs. 57.3%, *p* value < 0.02), and younger age at first birth (22.4 vs. 26.9 years, *p* value = 0.004). In comparison with high family income group, low family income group women were also less likely to complete college or attain postgraduate education (48.1% vs. 80.9%, *p* value < 0.001). Lastly, there was much higher portion of Black women in low family income group than in relatively higher family income group (73.5% vs. 38.2%, *p* value < 0.001).

**Table 1:** Descriptive characteristics of breast cancer patients in WCHS with methylation data

	Income (≤ 50,000) *n* = 245	Income (> 50,000) *n* = 314
Age at diagnosis		
Mean ± std	52.8 ± 11.4	50.5 ± 9.8
45 below	63 (25.7)	85 (27.1)
45–55	73 (29.8)	125 (39.8)
56–65	81 (33.1)	86 (27.4)
66–75	28 (11.4)	18 (5.7)
BMI (kg/m^2^)^a^		
Mean ± std	29.6 ± 8.3	27.5 ± 6.9
< 25.0 (normal)	54 (22.7)	115 (37.1)
25–29.9 (overweight)	73 (30.7)	97 (31.3)
≥ 30.0 (obesity)	111 (46.6)	98 (31.6)
Menopausal status		
Yes	108 (44.1)	180 (57.3)
No	137 (55.9)	134 (42.7)
Family history		
Yes	40 (16.3)	61 (19.4)
No	205 (83.7)	253 (80.6)
Age at first birth^b^		
Mean ± std	22.4 ± 5.6	26.9 ± 6.1
20 below	68 (35.6)	25 (11.3)
20–30	100 (52.4)	128 (57.9)
30–40	22 (11.5)	66 (29.9)
40 above	1 (0.5)	2 (0.9)
ER status		
Negative	62 (25.3)	67 (21.3)
Positive	183 (74.7)	247 (78.7)
Race		
AA	180 (73.5)	120 (38.2)
EA	65 (26.5)	194 (61.8)
Education		
Below high school	30 (12.2)	3 (0.9)
High school or equal	97 (39.6)	57 (18.2)
College	62 (25.3)	61 (19.4)
College graduate	42 (17.1)	106 (33.8)
Postgraduate and above	14 (5.7)	87 (27.7)

WCHS, Women’s Circle of Health Study; BMI, body mass index; AA, African-American; EA, European American

^a^7 cases in low family income group missing BMI information and 4 cases in higher family income group missing BMI information

^b^54 cases in low family income group missing age at first birth information and 93 cases in higher family income group missing age at first birth information

### Breast tumor DNA methylation with socioeconomic status

**Fig. 1:**
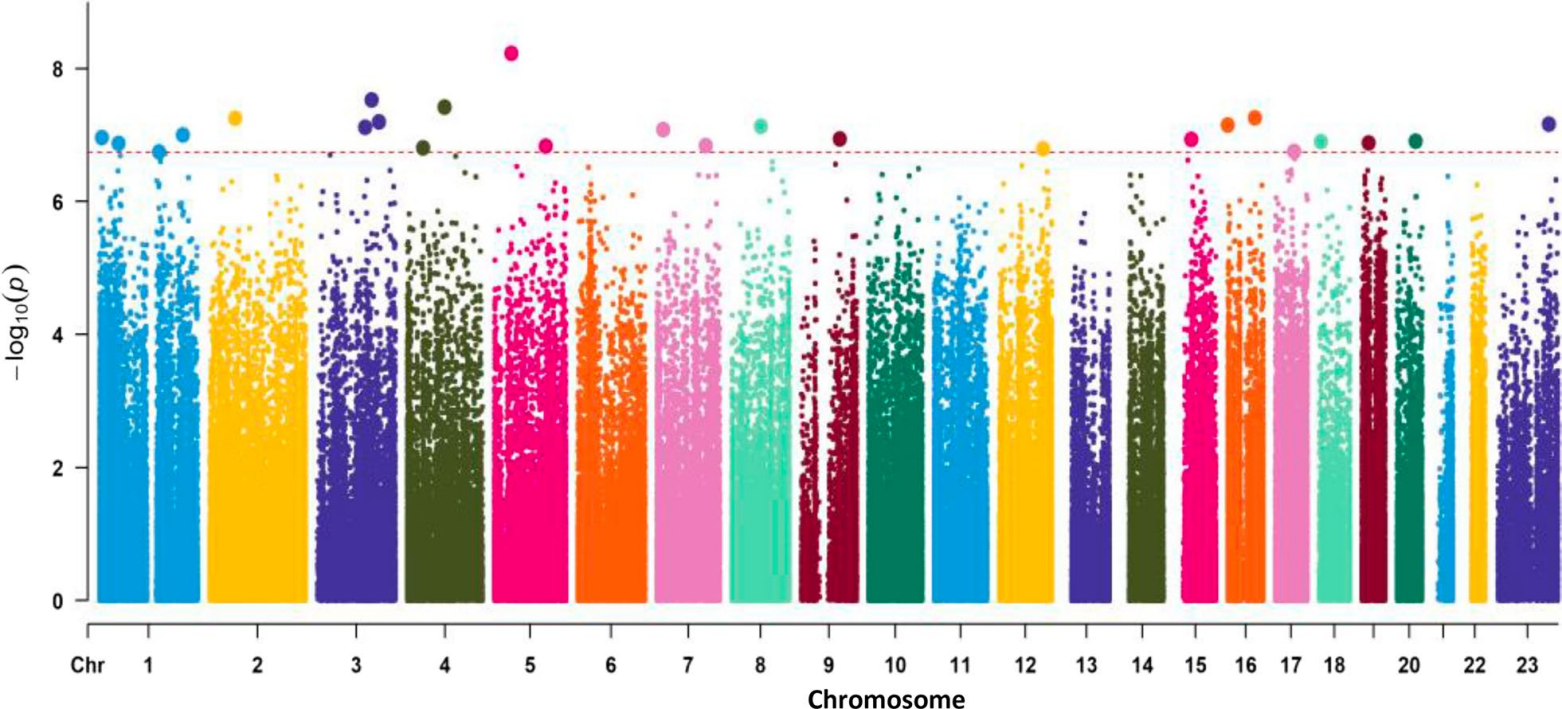
Manhattan plot for epigenome-wide associations of family income and breast tumor DNA methylation. Linear regression between family income (2-level) and DNA methylation levels was assessed, adjusting for age, race, and tumor estrogen receptor (ER) status. *Bonferroni* corrections were used to adjust for multiple comparison. The adjusted *p* values were log-transformed, negated, and plotted based on chromosomal position. The red horizontal line indicates the cutoff significance level of 1.8e−7

In the analysis of household income (> $50,000 vs. ≤ $50,000) in relation to DNA methylation, 25 CpG sites reached Bonferroni significance (*p* value = 1.8e−7) after adjusting for age, race, and ER status (Fig. 1). In contrast, no CpG site was associated with educational attainment (college and above vs. no college, Additional file 1: Fig. S1). To examine linear “dose–response” between household income and tumor DNA methylation levels, a 5-level income was tested, and the results, together with those from the 2-level income tests, are summarized in Table 2. The direction of associations with all the 25 CpG sites remained consistent, three of which remained array-wide significant, including cg00452016 in *nicotinamide nucleotide transhydrogenase* (*NNT*), cg04990372 in *MIR1259*, and cg01667837 in *G protein-coupled receptor 37* (*GPR37*). After further adjustment for study sites, 5 of the 25 CpG sites were associated with the 2-level income at the array-wide significance level, although all of the associations remained in the same direction with similar effect size (Additional file 5: Table S1).

**Table 2:** CpG loci exhibit significant association between family income and breast tumor DNA methylation

	Coefficient (2-levels)	*p* value (2-levels)	Coefficient (5-levels)	*p* value (5-levels)	Chr	MapInfo	Gene in proximity	Genomic Features
**cg00452016**	0.26	5.92E−09	0.08	5.50E−08	5	43603138	*NNT*	5′UTR
cg14216142	0.22	2.98E−08	0.05	1.77E−05	3	138313050	*CEP70*	5′UTR
cg08962709	0.14	3.81E−08	0.04	8.67E−07	4	95128673	*SMARCAD1*	TSS1500
cg10319857	− 0.05	5.53E−08	− 0.01	1.09E−06	16	69653345	*NFAT5*	5′UTR
cg05411428	0.17	5.57E−08	0.05	5.26E−06	2	64751054	*AFTPH*	TSS1500
cg08879724	0.17	6.36E−08	0.05	1.11E−06	3	156893035		
cg01225298	0.08	6.91E−08	0.03	2.38E−07	X	132091287	*HS6ST2*	Body
cg00547727	− 0.05	7.12E−08	− 0.01	2.16E−06	16	714968	*WDR90*	Body
cg06060338	0.21	7.41E−08	0.06	9.61E−07	8	74884601	*TCEB1*	TSS1500
cg19935671	0.11	7.62E−08	0.03	1.01E−06	3	121468093	*GOLGB1*	5′UTR
cg23402769	0.15	8.28E−08	0.05	2.07E−07	7	16685581	*BZW2*	TSS200
cg16262415	0.11	1.00E−07	0.04	2.02E−07	1	211848604	*NEK2*	Body
cg12302560	− 0.06	1.09E−07	− 0.02	5.71E−06	1	6611319	*NOL9*	Body
cg05176996	0.21	1.14E−07	0.06	1.58E−06	9	98637794	*C9orf130*	TSS200
cg02847760	0.11	1.16E−07	0.03	3.09E−06	15	40453199	*BUB1B*	TSS200
**cg04990372**	0.11	1.24E−07	0.04	1.23E−08	20	47895899	*MIR1259*	TSS1500
cg14291066	0.15	1.26E−07	0.05	4.11E−07	18	5238200	*C18orf18*	TSS200
cg03170171	− 0.06	1.33E−07	− 0.02	1.11E−05	19	17389968	*C19orf62*	3′UTR
cg12051027	− 0.06	1.34E−07	− 0.02	1.95E−06	1	48931095	*SPATA6*	Body
**cg01667837**	0.16	1.45E−07	0.05	1.62E−07	7	124405605	*GPR37*	5′UTR
cg10838757	0.18	1.47E−07	0.05	5.99E−06	5	130507273	*LYRM7*	Body
cg20359202	− 0.05	1.56E−07	− 0.01	3.05E−06	4	40626144	*RBM47*	5′UTR
cg00391320	− 0.05	1.62E−07	− 0.02	2.04E−07	12	112127825	*ACAD10*	5′UTR
cg20705065	0.11	1.77E−07	0.03	1.91E−06	17	48074613		
cg06526960	− 0.03	1.79E−07	− 0.01	1.18E−05	1	151336795	*SELENBP1*	3′UTR

Linear regression was performed using *limma* to test associations between DNA methylation levels at each CpG site and family income levels (2- or 5-levels), adjusted for age at diagnosis (continuous values), race (Black vs. White), and tumor estrogen receptor (ER) status (positive vs. negative)

**Fig. 2:**
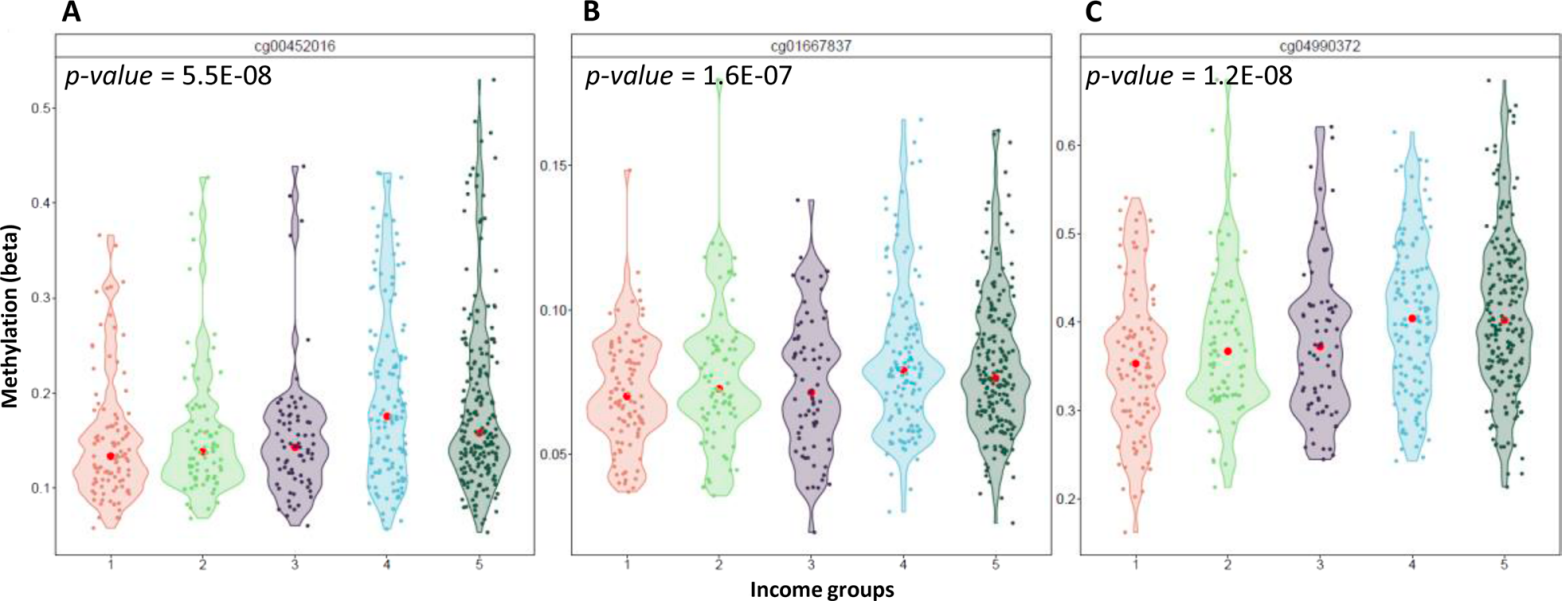
Top 3 CpG loci from association analysis between 5-level family income and breast tumor DNA methylation. Linear regression was performed, adjusting for age at diagnosis, race, and tumor estrogen receptor (ER) status. The adjusted *p* values are shown. **A** cg00452016 close to *NNT*; **B** cg01667837 close to *GPR37*; **C** cg04990372 close to *MIR1259*

**Fig. 3:**
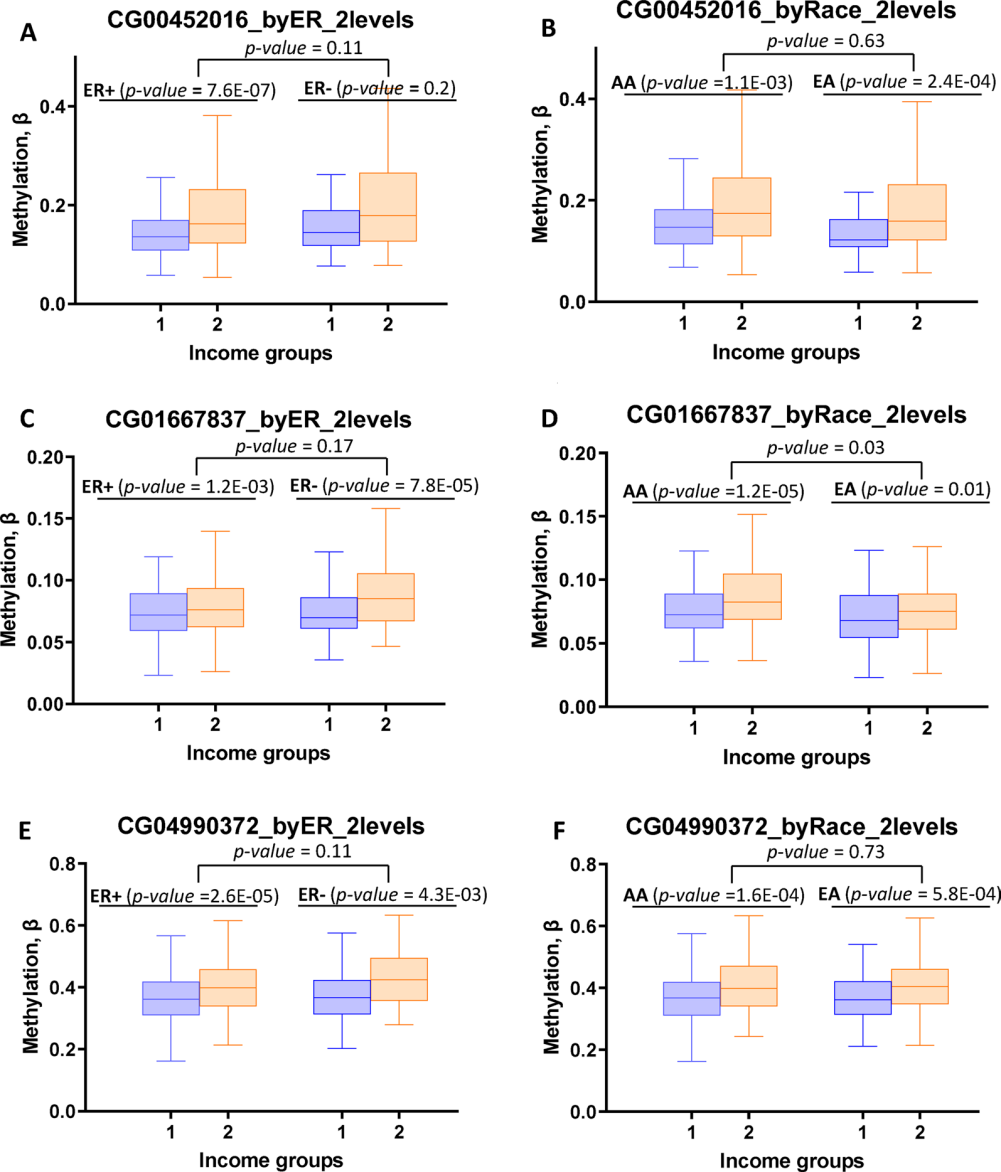
Differential methylation levels for cg00452016 (**A**, **B**), cg01667837 (**C**, **D**), and cg04990372 (**E**, **F**) between dichotomized income groups. For each of the CpG sites, linear regression was used to test association between income levels and DNA methylation within estrogen receptor (ER)+ or ER− subgroups, or within Black or White subgroups. Regression models were adjusted for age at diagnosis, and race (when stratified by ER status) or ER status (when stratified by race). The adjusted *p* values are shown. The average methylation level differences between the two ethnic groups and the two ER status groups were also tested using Mann–Whitney *U* test and the *p* values are labeled on top

When the analyses of the 2-level income were stratified by race or tumor ER status, the results did not differ between the subgroups, with the associations remained in the same direction, although some of the findings in White or ER-negative groups were not statistically significant (*p* value > 0.05), possibly due to smaller sample size (Additional file 5: Tables S2 and S3). As shown in Fig. 2, the methylation levels of the three array-wide significant CpG sites were higher among those with higher income levels. The trends were again similar between Black and White subgroups, as well as between ER-positive and ER-negative subtypes (Fig. 3), although the difference in methylation status of cg00452016 was not significant in ER-negative subtype.

### Functional exploration of SEP-associated CpG methylation

In publicly accessible data from TCGA breast cancer cohort, the methylation levels of cg00452016 in *NNT* (rho = -0.16, *p* value = 2.6e−5) and cg01667837 in *GPR37* (rho = -0.19, *p* value = 6.3e−7) were inversely correlated with their corresponding gene expression (Additional file 2: Fig. S2). Additional file 3: Fig. S3 and Additional file 4: Fig. S4 show the regulatory features of the genomic regions where cg00452016 and cg01667837 reside, respectively. Both regions contain multiple *cis*-elements consisting of active promoter of the chromHMM state, DNaseI hypersensitive sites, suggesting multiple *cis*-epigenetic regulatory mechanisms in these regions.

## Discussion

In an epigenome-wide analysis of SEP factors with DNA methylation in breast tumors, we identified 25 CpG sites across the genome associated with total household income, but none with educational attainment. Two of the top CpG sites, cg00452016 and cg01667837 located in the promoter region of *NNT* and *GPR37,* respectively, are of particular interest for the multiple epigenetic regulatory features in these regions, their inverse correlations with mRNA expression in breast tumor tissues, and moreover, their putative biological functions in mediating the biological impact of SEP.

One top hit in our association analysis was mapped to *NNT*, which encodes an enzyme playing crucial roles in maintaining mitochondrial redox balance in a wide variety of organs including the brain. Defective *NNT* function has been implicated in heart failure [27], neurodegenerative diseases [28], and a rare congenital condition known as familial glucocorticoid deficiency (FGD) [29]. FGD is characterized by an inability of the adrenal cortex to produce cortisol, a key stress hormone molecule that could be triggered by chronic socioeconomic stress in patients from the low SEP group [30]. Herein, cortisol mediates β-adrenergic stress signaling and inflammatory responses within individuals of lower SEP, explaining increased susceptibility to chronic diseases [31]. Thus, income-dependent methylation of *NNT* might blunt the rising cortisol levels and chronic inflammation that would otherwise be observed in the lower income group.

Another top hit, *GPR37*, is an orphan G protein-coupled receptor found with high abundance in the central nervous system (CNS). Deregulated *GPR37*-mediated G protein linked signaling pathway has been implicated in neurological disorders including Parkinson’s disease and autism spectrum disorder [32, 33], as well as psychiatric diseases including bipolar disorder (BPD) and major depression disorder (MDD) [34]. Interestingly, GPR37 levels were decreased in MDD but increased in BPD. *Gpr37*-knockout female mice showed significantly increased anxiety and depression-like behaviors [35]. Outside the CNS, *GPR37* was found in macrophages in the immune system. Activation of *GPR37* promotes macrophage phagocytosis and clearance of pathogens [36], and *Gpr37*-deficient mice showed delayed resolution of inflammatory pain featured by dysregulation of proinflammatory and anti-inflammatory cytokines [37]. Aberrant expression of *GPR37* was also implicated in several cancers, including gastric cancer, multiple myeloma, and hepatocellular carcinoma [38–41], yet little is known of its involvement in breast cancer. Interestingly, recent studies have demonstrated increased β-adrenergic autonomous sympathetic neuronal activities and their immunosuppression effects in breast cancer. Because GPR37 is a receptor for prosaposin [42], a potent neurotrophic factor with activities in inhibition of neuron apoptosis and promoting neurite outgrowth and neuron regeneration [43], *GPR37* might be involved in transducing β-adrenergic signaling in response to chronic psychosocial stress in breast tumor tissues. In patients with low household income, demethylation of *GPR37* and subsequent increased expression as found in our study might lead to elevated β-adrenergic stress signaling and immunosuppression in the tumor microenvironment.

As lower SEP might lead to chronic psychosocial stress [1], we hypothesized that it could impact cancer development and progression through two biological pathways: alterations in β-adrenergic stress response and immunosuppression [44, 45]. Long-term and continuous stress disturbs the neuroendocrine balance through the hypothalamic–pituitary–adrenal axis, perpetuating a state of psychological and physiological ‘burnout’. Chronic stress also causes high levels of chronic inflammation and suppresses different facets of immune functions, creating a pro-cancer immunological milieu [46]. Our agnostic epigenome-wide analysis now provides new evidence to suggest that stress-response and immunosuppression pathways might be important in understanding how socioeconomic status impacts tumor development and progression.

It should be noted that family income and educational attainment used in our analysis are only two crude proxies of psychosocial stressors, and by no means the only measures in terms of the scope, depth and complexity of stressors from various sources. They may not reflect the actual perceived stress or individual coping capability, either. Nevertheless, the identification of *NNT* and *GPR37* in our study lends support to the validity of household income as a commonly available measure of socioeconomic stress. Although we set out to perform an epigenome-wide analysis without a priori hypothesis, the two most significant genes came from stress-related neuroendocrine response and immune response pathways, the two central biological mechanisms that were previously proposed [44, 45]. This highlights the importance of these two pathways to regulate the biological effects of psychosocial stress. Moreover, the associations of family income and DNA methylation at these two CpG sites appeared to be linear, consistent with the previous findings of a graded association between SEP and health, where each improvement in education, income, occupation, or wealth was associated with better health outcomes [47].

Other limitations of this study include a relatively small sample size that would not allow us to perform separate analysis by race or tumor subtypes, the use of the 450 K methylation array instead of the newer EPIC assay or methylation sequencing, and the one-time assessment of SEP and DNA methylation. It is also important to note that the fold change of methylation status of the CpG sites associated with family income in our study was much smaller than those by tumor vs. normal or by tumor ER status. Future studies are warranted to replicate our findings and to investigate the downstream biological and clinical significance of the methylation changes and whether the findings can be extrapolated to other cancer types.

In conclusion, in a racially diverse breast cancer patient population, we discovered evidence of biological impact of household income on tumor DNA methylation, particularly on genes involved in the β-adrenergic stress and immune response pathways. Because most previous socioepigenomic studies relied on blood samples to assess the systemic impact of socioeconomic status with indirect relevance to local tumor tissues, our new findings support biological effects on tumor tissues by SEP, which are likely to modulate cancer development and progression.

## Supplementary Information

**Additional file 1: Fig. S1.** Manhattan plot for epigenome-wide associations of educational attainment and breast tumor DNA methylation. Linear regression between education (2-level) and DNA methylation levels were assessed using *limma* package after adjusting age, race, and estrogen receptor status. *Bonferroni* corrections were used to adjust for multiple comparison. The adjusted *p* values were log-transformed, negated, and plotted based on chromosomal position. The red horizontal line indicates the cutoff significance level of 1.8e−7.

**Additional file 2: Fig. S2.** Inverse correlation between DNA methylation and gene expression levels in the TCGA BRCA cohorts (780 participants) for cg00452016/*NNT* (**A**) and cg01667837/*GPR37* (**B**). Adjusted *p* values from Spearman test are shown.

**Additional file 3: Fig. S3.** Integrative annotation of molecular features of the genomic regions of cg00452016/*NNT*. From top to bottom, the tracks show: *NNT*; the cg00452016 site highlighted in vertical cyan line; measurements of evolutionary conservation from alignments of 100 vertebrate species; conserved transcriptional binding sites; the hypersensitive DNaseI sites profile of HMEC and MCF cell lines of the ENCODE project; the HMEC chromHMM tracks indicating putative active (bright red) promoters, strong enhancer (orange), strong transcript (green), as well as putative weak enhancers (yellow); histone modifications of H3K27ac, H3K4me1/3 in various breast cell types.

**Additional file 4: Fig. S4.** Integrative annotation of molecular features of the genomic regions of cg01667837/*GPR37*. From top to bottom, the tracks showing: *GPR37*; the cg01667837 site highlighted in vertical cyan line; measurements of evolutionary conservation from alignments of 100 vertebrate species; conserved transcriptional binding sites; the hypersensitive DNaseI sites profile of HMEC and MCF cell lines of the ENCODE project; the HMEC chromHMM tracks indicating putative active (bright red) promoters, strong enhancer (orange), strong transcript (green), as well as putative weak enhancers (yellow); histone modifications of H3K27ac, H3K4me1/3 in various breast cell types.

**Additional file 5. Supplemental Table S1.** CpG loci exhibit significant association between family income and breast tumor DNA methylation. Linear regression was performed using limma to test associations between DNA methylation levels at each CpG site and family income levels (2- or 5-levels), adjusted for age at diagnosis (continuous values), race (Black vs. White), recruitment sites, and tumor estrogen receptor (ER) status (positive vs. negative). **Supplemental Table S2**. Associations between family income and methylation levels at significant CpG sites by race. Linear regression was performed using limma to test associations between DNA methylation levels at each CpG site and family income levels (2-levels) in Black and White subgroups separately, adjusted for age at diagnosis (continuous values), recruitment sites, and tumor estrogen receptor (ER) status (positive vs. negative). **Supplemental Table S3**. Associations between family income and methylation levels at significant CpG sites by tumor estrogen receptor status. Linear regression was performed using limma to test associations between DNA methylation levels at each CpG site and family income levels family income levels (2-levels) in estrogen receptor (ER) status (positive and negative) subgroups separately, adjusted for age at diagnosis (continuous values), recruitment sites, and race (Black vs. White).

## Data Availability

DNA methylation data and phenotype data analyzed in this study have been deposited in GEO under accession number GSE226569.
