# Supplementary material for: An epigenome-wide analysis of socioeconomic position and tumor DNA methylation in breast cancer patients
Source: Clin Epigenetics. 2023 Apr 26;15:68. doi: 10.1186/s13148-023-01470-4 (PMC10131486; doi:10.1186/s13148-023-01470-4)
Supplement: Supplementary file 5 — Additional file 5. Supplemental Table S1. CpG loci exhibit significant association between family income and breast tumor DNA methylation. Linear regression was performed using limma to test associations between DNA methylation levels at each CpG site and family income levels (2- or 5-levels), adjusted for age at diagnosis (continuous values), race (Black vs. White), recruitment sites, and tumor estrogen receptor (ER) status (positive vs. negative). Supplemental Table S2. Associations between family income and methylation levels at significant CpG sites by race. Linear regression was performed using limma to test associations between DNA methylation levels at each CpG site and family income levels (2-levels) in Black and White subgroups separately, adjusted for age at diagnosis (continuous values), recruitment sites, and tumor estrogen receptor (ER) status (positive vs. negative). Supplemental Table S3. Associations between family income and methylation levels at significant CpG sites by tumor estrogen receptor status. Linear regression was performed using limma to test associations between DNA methylation levels at each CpG site and family income levels family income levels (2-levels) in estrogen receptor (ER) status (positive and negative) subgroups separately, adjusted for age at diagnosis (continuous values), recruitment sites, and race (Black vs. White). [file 13148_2023_1470_MOESM5_ESM.docx]

**Supplemental Table S1. CpG loci exhibit significant association between family income and breast tumor DNA methylation with additional adjustment for recruitment site**

|  | **Coefficient (2-levels)** | **p-value (2-levels)** | **Coefficient (5-levels)** | **p-value (5-levels)** | **Chr** | **MapInfo** | **Gene in proximity** | **Genomic Features** |
| --- | --- | --- | --- | --- | --- | --- | --- | --- |
| **cg00452016** | 0.24 | 1.57E-07 | 0.07 | 1.21E-06 | 5 | 43603138 | *NNT* | 5'UTR |
| cg14216142 | 0.22 | 8.62E-08 | 0.05 | 4.71E-05 | 3 | 138313050 | *CEP70* | 5'UTR;1stExon |
| cg08962709 | 0.13 | 3.61E-07 | 0.04 | 6.97E-06 | 4 | 95128673 | *SMARCAD1* | TSS1500 |
| cg10319857 | -0.04 | 6.95E-07 | -0.01 | 1.13E-05 | 16 | 69653345 | *NFAT5* | 5'UTR;Body |
| cg05411428 | 0.16 | 5.98E-07 | 0.04 | 4.19E-05 | 2 | 64751054 | *AFTPH* | TSS1500 |
| cg08879724 | 0.16 | 6.34E-07 | 0.05 | 9.29E-06 | 3 | 156893035 |  |  |
| cg01225298 | 0.08 | 9.97E-08 | 0.03 | 3.57E-07 | X | 132091287 | *HS6ST2* | Body |
| cg00547727 | -0.05 | 1.17E-07 | -0.01 | 3.82E-06 | 16 | 714968 | *WDR90* | Body |
| cg06060338 | 0.20 | 3.43E-07 | 0.06 | 4.35E-06 | 8 | 74884601 | *TCEB1* | TSS1500 |
| cg19935671 | 0.10 | 4.43E-07 | 0.03 | 5.08E-06 | 3 | 121468093 | *GOLGB1* | 5'UTR |
| cg23402769 | 0.14 | 7.93E-07 | 0.04 | 1.79E-06 | 7 | 16685581 | *BZW2* | TSS200 |
| cg16262415 | 0.10 | 1.28E-06 | 0.03 | 2.35E-06 | 1 | 211848604 | *NEK2* | Body |
| cg12302560 | -0.06 | 3.19E-07 | -0.02 | 1.69E-05 | 1 | 6611319 | *NOL9* | Body |
| cg05176996 | 0.20 | 6.86E-07 | 0.06 | 8.63E-06 | 9 | 98637794 | *C9orf130* | TSS200;Body |
| cg02847760 | 0.11 | 3.41E-07 | 0.03 | 8.76E-06 | 15 | 40453199 | *BUB1B* | TSS200 |
| **cg04990372** | 0.12 | 1.40E-07 | 0.04 | 1.36E-08 | 20 | 47895899 | *MIR1259* | TSS1500;Body |
| cg14291066 | 0.14 | 1.42E-06 | 0.04 | 4.16E-06 | 18 | 5238200 | *C18orf18* | TSS200;Body |
| cg03170171 | -0.05 | 7.92E-07 | -0.02 | 5.66E-05 | 19 | 17389968 | *C19orf62* | 3'UTR |
| cg12051027 | -0.06 | 5.44E-07 | -0.02 | 7.75E-06 | 1 | 48931095 | *SPATA6* | Body |
| **cg01667837** | 0.16 | 2.88E-07 | 0.05 | 3.36E-07 | 7 | 124405605 | *GPR37* | 1stExon;5'UTR |
| cg10838757 | 0.18 | 4.27E-07 | 0.05 | 1.69E-05 | 5 | 130507273 | *LYRM7* | Body |
| cg20359202 | -0.05 | 1.14E-06 | -0.01 | 1.94E-05 | 4 | 40626144 | *RBM47* | 5'UTR |
| cg00391320 | -0.05 | 2.34E-07 | -0.02 | 3.17E-07 | 12 | 112127825 | *ACAD10* | 5'UTR |
| cg20705065 | 0.10 | 1.51E-06 | 0.03 | 1.46E-05 | 17 | 48074613 |  |  |
| cg06526960 | -0.03 | 4.56E-07 | -0.01 | 2.99E-05 | 1 | 151336795 | *SELENBP1* | 3'UTR |

***Footnote:*** Linear regression was performed using *limma* to test associations between DNA methylation levels at each CpG site and family income levels (2- or 5-levels), adjusted for age at diagnosis (continuous values), race (Black vs. White), recruitment sites, and tumor estrogen receptor (ER) status (positive vs. negative).

**Supplemental Table S2. Associations between family income and breast tumor methylation levels at significant CpG sites by race**

| **CpG probe** | **Coefficient (Black)** | **Raw p (Black)** | **Coefficient (White)** | **Raw p (White)** | **Chr** | **MapInfo** | **Gene in proximity** | **Genomic Features** |
| --- | --- | --- | --- | --- | --- | --- | --- | --- |
| **cg00452016** | 0.22 | 9.11E-05 | 0.32 | 1.99E-05 | 5 | 43603138 | NNT | 5'UTR |
| cg14216142 | 0.22 | 9.65E-06 | 0.23 | 6.71E-04 | 3 | 1.38E+08 | CEP70 | 5'UTR |
| cg08962709 | 0.15 | 1.09E-06 | 0.11 | 5.25E-03 | 4 | 95128673 | SMARCAD1 | TSS1500 |
| cg10319857 | -0.04 | 1.66E-04 | -0.05 | 9.70E-05 | 16 | 69653345 | NFAT5 | 5'UTR |
| cg05411428 | 0.2 | 1.26E-06 | 0.14 | 8.03E-03 | 2 | 64751054 | AFTPH | TSS1500 |
| cg08879724 | 0.17 | 4.25E-05 | 0.17 | 5.12E-04 | 3 | 1.57E+08 |  |  |
| cg01225298 | 0.08 | 9.12E-06 | 0.08 | 1.81E-03 | X | 1.32E+08 | HS6ST2 | Body |
| cg00547727 | -0.05 | 1.31E-05 | -0.04 | 2.14E-03 | 16 | 714968 | WDR90 | Body |
| cg06060338 | 0.18 | 1.83E-04 | 0.23 | 1.85E-04 | 8 | 74884601 | TCEB1 | TSS1500 |
| cg19935671 | 0.12 | 2.30E-06 | 0.08 | 9.54E-03 | 3 | 1.21E+08 | GOLGB1 | 5'UTR |
| cg23402769 | 0.15 | 2.21E-05 | 0.14 | 1.97E-03 | 7 | 16685581 | BZW2 | TSS200 |
| cg16262415 | 0.11 | 2.69E-05 | 0.11 | 1.05E-03 | 1 | 2.12E+08 | NEK2 | Body |
| cg12302560 | -0.06 | 2.80E-05 | -0.06 | 2.12E-03 | 1 | 6611319 | NOL9 | Body |
| cg05176996 | 0.2 | 8.02E-05 | 0.21 | 6.20E-04 | 9 | 98637794 | C9orf130 | TSS200 |
| cg02847760 | 0.13 | 8.21E-07 | 0.07 | 2.91E-02 | 15 | 40453199 | BUB1B | TSS200 |
| **cg04990372** | 0.11 | 1.30E-04 | 0.12 | 2.99E-04 | 20 | 47895899 | MIR1259 | TSS1500 |
| cg14291066 | 0.19 | 1.16E-06 | 0.1 | 2.29E-02 | 18 | 5238200 | C18orf18 | TSS200 |
| cg03170171 | -0.06 | 3.36E-05 | -0.05 | 2.29E-03 | 19 | 17389968 | C19orf62 | 3'UTR |
| cg12051027 | -0.06 | 1.35E-04 | -0.06 | 3.26E-04 | 1 | 48931095 | SPATA6 | Body |
| **cg01667837** | 0.17 | 1.89E-05 | 0.16 | 9.52E-04 | 7 | 1.24E+08 | GPR37 | 5'UTR |
| cg10838757 | 0.24 | 3.36E-06 | 0.11 | 1.37E-02 | 5 | 1.31E+08 | LYRM7 | Body |
| cg20359202 | -0.05 | 1.55E-04 | -0.05 | 5.38E-04 | 4 | 40626144 | RBM47 | 5'UTR |
| cg00391320 | -0.05 | 2.36E-05 | -0.05 | 2.32E-03 | 12 | 1.12E+08 | ACAD10 | 5'UTR |
| cg20705065 | 0.11 | 5.31E-05 | 0.11 | 1.15E-03 | 17 | 48074613 |  |  |
| cg06526960 | -0.03 | 4.22E-05 | -0.03 | 2.16E-03 | 1 | 1.51E+08 | SELENBP1 | 3'UTR |

***Footnote:*** Linear regression was performed using *limma* to test associations between family income (2-levels) and DNA methylation levels at each CpG site in Black and White subgroups separately, adjusted for age at diagnosis (continuous values) and tumor estrogen receptor (ER) status (positive vs. negative).

**Supplemental Table S3. Associations between family income and breast tumor methylation levels at significant CpG sites by tumor estrogen receptor status**

|  | **Coefficient (ER-)** | **Raw p (ER-)** | **Coefficient (ER+)** | **Raw p (ER+)** | **Chr** | **MapInfo** | **Gene in proximity** | **Genomic Features** |
| --- | --- | --- | --- | --- | --- | --- | --- | --- |
| **cg00452016** | 0.22 | 0.016 | 0.27 | 1.41E-07 | 5 | 43603138 | NNT | 5'UTR |
| cg14216142 | 0.19 | 0.008 | 0.23 | 1.27E-06 | 3 | 1.38E+08 | CEP70 | 5'UTR |
| cg08962709 | 0.2 | 0 | 0.12 | 4.05E-05 | 4 | 95128673 | SMARCAD1 | TSS1500 |
| cg10319857 | -0.04 | 0.038 | -0.05 | 5.24E-07 | 16 | 69653345 | NFAT5 | 5'UTR |
| cg05411428 | 0.22 | 0 | 0.16 | 2.60E-05 | 2 | 64751054 | AFTPH | TSS1500 |
| cg08879724 | 0.19 | 0.002 | 0.16 | 8.40E-06 | 3 | 1.57E+08 |  |  |
| cg01225298 | 0.11 | 0.001 | 0.07 | 2.10E-05 | X | 1.32E+08 | HS6ST2 | Body |
| cg00547727 | -0.05 | 0.007 | -0.05 | 4.14E-06 | 16 | 714968 | WDR90 | Body |
| cg06060338 | 0.23 | 0.004 | 0.2 | 8.89E-06 | 8 | 74884601 | TCEB1 | TSS1500 |
| cg19935671 | 0.08 | 0.043 | 0.12 | 4.76E-07 | 3 | 1.21E+08 | GOLGB1 | 5'UTR |
| cg23402769 | 0.11 | 0.041 | 0.16 | 6.88E-07 | 7 | 16685581 | BZW2 | TSS200 |
| cg16262415 | 0.17 | 0 | 0.1 | 8.02E-05 | 1 | 2.12E+08 | NEK2 | Body |
| cg12302560 | -0.05 | 0.017 | -0.06 | 2.70E-06 | 1 | 6611319 | NOL9 | Body |
| cg05176996 | 0.18 | 0.022 | 0.22 | 1.49E-06 | 9 | 98637794 | C9orf130 | TSS200 |
| cg02847760 | 0.12 | 0.01 | 0.1 | 3.75E-06 | 15 | 40453199 | BUB1B | TSS200 |
| **cg04990372** | 0.15 | 0.001 | 0.1 | 3.03E-05 | 20 | 47895899 | MIR1259 | TSS1500 |
| cg14291066 | 0.13 | 0.021 | 0.16 | 2.43E-06 | 18 | 5238200 | C18orf18 | TSS200 |
| cg03170171 | -0.05 | 0.014 | -0.06 | 3.20E-06 | 19 | 17389968 | C19orf62 | 3'UTR |
| cg12051027 | -0.07 | 0.006 | -0.06 | 8.52E-06 | 1 | 48931095 | SPATA6 | Body |
| **cg01667837** | 0.26 | 0 | 0.13 | 0.000252 | 7 | 1.24E+08 | GPR37 | 5'UTR |
| cg10838757 | 0.21 | 0.003 | 0.18 | 1.07E-05 | 5 | 1.31E+08 | LYRM7 | Body |
| cg20359202 | -0.05 | 0.018 | -0.05 | 3.71E-06 | 4 | 40626144 | RBM47 | 5'UTR |
| cg00391320 | -0.05 | 0.011 | -0.05 | 6.14E-06 | 12 | 1.12E+08 | ACAD10 | 5'UTR |
| cg20705065 | 0.1 | 0.017 | 0.12 | 3.59E-06 | 17 | 48074613 |  |  |
| cg06526960 | -0.02 | 0.221 | -0.04 | 1.74E-07 | 1 | 1.51E+08 | SELENBP1 | 3'UTR |

***Footnote:*** Linear regression was performed using *limma* to test associations between DNA methylation levels at each CpG site and family income levels (2-levels) in Black and White subgroups separately, adjusted for age at diagnosis (continuous values) and ethnicity (Black vs. White).
